# Gas Partial Pressure in Cultured Cells: Patho-Physiological Importance and Methodological Approaches

**DOI:** 10.3389/fphys.2018.01803

**Published:** 2018-12-13

**Authors:** Ramon Farré, Isaac Almendros, Josep M. Montserrat, David Gozal, Daniel Navajas

**Affiliations:** ^1^Unitat de Biofísica i Bioenginyeria, Facultat de Medicina i Ciències de la Salut, Universitat de Barcelona, Barcelona, Spain; ^2^CIBER de Enfermedades Respiratorias, Madrid, Spain; ^3^Institut d’Investigacions Biomèdiques August Pi i Sunyer, Barcelona, Spain; ^4^Sleep Lab, Hospital Clinic of Barcelona, Barcelona, Spain; ^5^Department of Child Health, University of Missouri School of Medicine, Columbia, MO, United States; ^6^Institute for Bioengineering of Catalonia, Barcelona Institute of Science and Technology, Barcelona, Spain

**Keywords:** hypoxia, hyperoxia, hypercapnia, cell microenvironment, respiratory diseases, sleep apnea, mechanical ventilation, cancer

## Abstract

Gas partial pressures within the cell microenvironment are one of the key modulators of cell pathophysiology. Indeed, respiratory gases (O_2_ and CO_2_) are usually altered in respiratory diseases and gasotransmitters (CO, NO, H_2_S) have been proposed as potential therapeutic agents. Investigating the pathophysiology of respiratory diseases *in vitro* mandates that cultured cells are subjected to gas partial pressures similar to those experienced by each cell type in its native microenvironment. For instance, O_2_ partial pressures range from ∼13% in the arterial endothelium to values as low as 2–5% in cells of other healthy tissues and to less than 1% in solid tumor cells, clearly much lower values than those used in conventional cell culture research settings (∼19%). Moreover, actual cell O_2_ partial pressure *in vivo* changes with time, at considerably different timescales as illustrated by tumors, sleep apnea, or mechanical ventilation. Unfortunately, the conventional approach to modify gas concentrations at the above culture medium precludes the tight and exact control of intra-cellular gas levels to realistically mimic the natural cell microenvironment. Interestingly, well-controlled cellular application of gas partial pressures is currently possible through commercially available silicone-like material (PDMS) membranes, which are biocompatible and have a high permeability to gases. Cells are seeded on one side of the membrane and tailored gas concentrations are circulated on the other side of the membrane. Using thin membranes (50–100 μm) the value of gas concentration is instantaneously (<0.5 s) transmitted to the cell microenvironment. As PDMS is transparent, cells can be concurrently observed by conventional or advanced microscopy. This procedure can be implemented in specific-purpose microfluidic devices and in settings that do not require expensive or complex technologies, thus making the procedure readily implementable in any cell biology laboratory. This review describes the gas composition requirements for a cell culture in respiratory research, the limitations of current experimental settings, and also suggests new approaches to better control gas partial pressures in a cell culture.

## Gas Partial Pressure: a Key Factor in Cell Microenvironment

Cell function is determined by the incessant chemical and physical cross-talk with its microenvironment. The fact that cell life critically depends on a series of molecular moieties within the tissue medium where the cell is located has been known since the early beginnings of biology as a science (Shaywitz and Melton, 2005). Indeed, our basic knowledge on cells has progressed and we now deeply understand the role played by the basic molecules involved in cell metabolism. However, it took longer to understand that other factors in the cell microenvironment are also highly relevant to cell physiology. For instance, we only recently realized that the extracellular matrix plays a fundamental role in cell biology, well beyond providing simple physical support within tissues (Piez, 1997).

Furthermore, a paradigm shift was required to understand that factors other than chemical species in the microenvironment were important modulators of cell function as well. Indeed, in the last decade we have gained clear insights to the importance of the physical microenvironment of the cell, specifically the mechanisms that we now include within the concept of mechanobiology (Iskratsch et al., 2014). Therefore, we currently know that both the 2D/3D architecture and the mechanical stiffness of the cell microenvironment are key cues for cell life and function (Wang B. et al., 2016). Accordingly, seeding cells on substrates with stiffness mimicking that of the *in vivo* tissues is clearly preferable and more biologically relevant than culturing cells on the non-physiological rigidity of plastic or glass. We also now understand that for some very specific cells (e.g., those within heart, lung, muscles, and bone) the dynamic mechanical microenvironment (e.g., tension, compression, cyclic stretch) as well as the static microenvironment modulates cell function, proliferation, differentiation, and migration (Roca-Cusachs et al., 2017; Uroz et al., 2018).

The progress in knowledge on the interaction between cells and their microenvironment achieved to date was made possible through the use of advanced concepts and technologies in disciplines such as genetics, proteomics, immunology, and biophysics. However, such intellectual investments aimed at discovering new mechanisms in cell pathophysiology contrasts with the relatively scant efforts devoted to the study of the effects of gases on cell functions, using relatively realistic experimental approaches, and more particularly in the case of the most fundamental gas, namely oxygen (Place et al., 2017). The role that O_2_ plays in cellular respiration has been known since the seminal work of Lavoisier in the 18th century (Underwood, 1944) and a great deal of sophistication has resulted in both the expansion in scope as well as the growth in biomedical research carried out since those early days (Prabhakar and Semenza, 2015).

Nevertheless, it is striking that most research in cultured cells, even when using the most advanced concepts and techniques, has been performed in experimental conditions that are far from physioxia, i.e., the normoxic level of cells within their natural environment (Carreau et al., 2011). Indeed, whereas the physiological partial pressures of O_2_ in cells range from a maximum of ∼13% in the arterial endothelium to values as low as 2–5% in cells of other normal tissues, and to less than 1% in tumor cells (Hunyor and Cook, 2018), cell biology and most pathophysiological mechanisms are usually investigated in culture chambers at ∼19% O_2_. Oxygenation in a conventional chamber is lower than room air (21% O_2_) because the partial pressure of the atmospheric N_2_–O_2_ gas mixture is reduced from ≈100% to ≈88.4% by externally imposing a 5% content of CO_2_ and a 6.2% content of water vapor (47 mmHg partial pressure at 37°C and saturation). The oxygen concentration in a conventional culture chamber is therefore ≈18.6% (i.e., 21% of 88.4%). It is remarkable that from a physiological viewpoint implementation of ∼19% O_2_ conditions clearly does not correspond to cellular normoxia, as claimed by most authors, but actually reflects considerable hyperoxia. Moreover, most studies aimed at studying the effects of hypoxia in cells have been carried out at 1–10% O_2_, which in fact leads to partial pressures close to physioxia (Carreau et al., 2011). It is of note that the interest of subjecting cultured cells to realistic gas concentrations corresponding to normal and diseases *in vivo* conditions refers not only to gases directly involved in respiration (O_2_ and CO_2_), but also encompasses other gases that are relevant from a pathophysiological and therapeutic viewpoint. Indeed, gasotransmitters, such as nitric oxide, carbon monoxide, and hydrogen sulfide (Wang, 2014), and hydrogen are all gases with unique biological properties that are critically dependent on their intracellular and extracellular concentrations.

This review is focused on discussing the importance of accurately controlling actual gas concentrations within the microenvironment of cultured cells, to realistically mimic normal and disease conditions *in vivo*, and will also describe the experimental procedures that are currently available to achieve more precise control of cellular gas milieu, particularly those techniques that can be implemented in a conventional cell biology laboratory.

## *In Vivo* Gas Partial Pressures Within the Cell Microenvironment

### Oxygen

Under normal conditions oxygen partial pressures vary considerably among the different tissues. Each type of cell is therefore normally subjected to its own homeostatic physiological level of oxygenation in healthy tissues (Carreau et al., 2011). Moreover, O_2_ probably plays the most relevant role in modulating cell, tissue, and patient responses in the context of different diseases. Indeed, most respiratory diseases are characterized by alterations of alveolar ventilation and/or gas diffusion capacity through the alveolar–capillary membrane, thereby resulting in different patterns of acute, chronic, and/or intermittent hypoxemia and thus tissue and cellular hypoxia. Although hypoxia is the most prevalent perturbation of O_2_ physioxia, it should be mentioned that hyperoxia is a potential risk for acute lung injury patients who are mechanically ventilated with high oxygen fraction (Damiani et al., 2014; Helmerhorst et al., 2015) and in newborns receiving critical care (Sola et al., 2014; Perrone et al., 2017). Depending on the disease, alterations in cell oxygenation are either continuous or are subjected to fluctuations with marked variations in temporal and amplitude domains. For example and to name just a few, infinite oscillation patterns are recorded in solid malignant tumors, hypoxic preconditioning, sleep apnea, or during mechanical ventilation in patients with acute respiratory failure.

In cancer, the degree of cell oxygenation in tumor cells varies considerably depending on their topographic location within the tumor (central vs. peripheral) (Chauhan et al., 2011; Nguyen et al., 2012). Moreover, given the abnormal angiogenesis processes taking place within tumors, any given cell may experience considerable O_2_ fluctuations over time, as tumor vessels remodel at fast rates, and perfusion patterns change (Kimura et al., 1996; Cárdenas-Navia et al., 2008; Matsumoto et al., 2010). Hypoxic pre-conditioning is a naturally occurring or a therapeutically used procedure based on modifying oxygenation levels using different time and cycle sequences aimed at attenuating the negative consequences of hypoxia/reoxygenation events (Anttila et al., 2016; Heyman et al., 2016; Rosenberg et al., 2018), such as in organ transplantation (Robertson et al., 2017), in cardiovascular diseases (Aimo et al., 2015; Heusch et al., 2015; Rosenberg et al., 2018), and in cerebral diseases and neuro-interventional procedures (Zhou et al., 2018).

**FIGURE 1:**
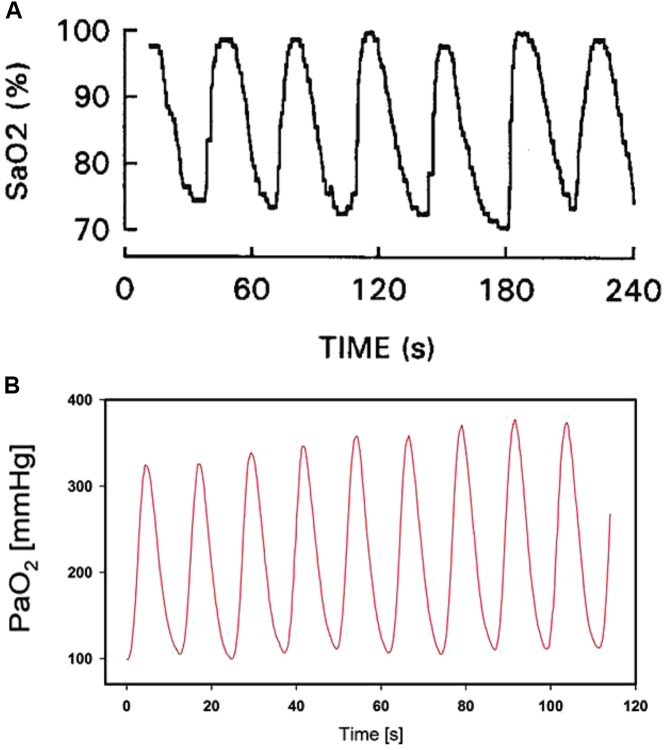
*In vivo* arterial blood oxygen levels in respiratory diseases. **(A)** Example of arterial oxygen saturation (SaO_2_) measured in a patient with obstructive sleep apnea. Reproduced from Farré et al. (1998) with permission of the copyright owner. **(B)** Example of oxygen partial pressure (PaO_2_) in the arterial blood induced by recruitment and de-recruitment in a pig with experimentally induced acute respiratory distress syndrome. Reproduced from Thomas et al. (2017) (http://creativecommons.org/licenses/by/4.0/).

As the rate of the oxygenation changes experienced by cells in cancer and hypoxic preconditioning is quite small (periods from 15 to 30 min to hours), the hypoxic–reoxygenation cycles are much faster in sleep apnea, reaching 60 events/h (i.e., one per minute) in patients with severe disease. Figure 1A shows a representative recording of arterial oxygen saturation in a patient with sleep apnea exhibiting fast and deep cycles of oxyhemoglobin desaturation (Farré et al., 1998). Such intermittent hypoxemia in sleep apnea is translated to virtually all patient tissues and organs, eliciting a considerable increase in the risk for morbidity and mortality. Extensive research in animal models of sleep apnea has shown that oxygen desaturations similar to those in severely diseased patients not only result in clear fast-rate oscillations of tissue O_2_ partial pressures in the brain, muscle, fat, liver, testis, and gut microbiota, but also illustrate that such oscillations are quite disparate across the various tissues, thereby requiring a very precise tailoring of the oxygenation profile in the case of cells from different organs, to mimic the actual *in vivo* situation (Almendros et al., 2011; Reinke et al., 2011; Torres et al., 2014; Moreno-Indias et al., 2015).

Recent research in the field of mechanical ventilation has revealed that under certain circumstances, cells in different tissues could be subjected to intermittent hyperoxia at cycling rates even higher than the one characterizing severe sleep apnea. Indeed, development of new ultrafast fiberoptic oxygen sensors (<200 μm in diameter), suitable for human use, can continually measure the variations of O_2_ partial pressure in blood throughout the breathing cycle (Formenti et al., 2015). It was recently shown that in healthy lungs, arterial oxygen partial pressure experiences marked respiratory oscillations depending on the conditions of mechanical ventilation (Formenti et al., 2017). These oxygen oscillations can reach much greater amplitudes in injured lungs subjected to mechanical ventilation with O_2_-enriched air and when experiencing alveolar opening and closure (cyclical atelectasis). Figure 1B shows an example of recruitment/de-recruitment-induced arterial blood O_2_ oscillations in a pig with acute lung injury (Thomas et al., 2017): O_2_ partial pressure swings ranged between 100 and 340 mmHg at a rate of 5 cycles/min (300 per hour, approximately fivefold faster than in severe sleep apnea). Importantly, such arterial oxygen oscillations are translated to tissues, e.g., the kidney (Thomas et al., 2017) and the brain (Klein et al., 2013), indicating that different cell types would be exposed to considerable intermittent oxidative stress.

In addition to these examples of diseases with well-known oxygen fluctuations, variable gas partial pressures could also be present in other pathologic conditions with respiratory instabilities, such as asthma (Kaminsky et al., 2017; Delgado-Eckert et al., 2018). Fluctuations in alveolar ventilation in asthmatic patients (Porra et al., 2018) could result in low-frequency intermittent hypoxia (and hypercapnia), although such gas partial pressures have so far not been well characterized. In this context, it is interesting to note that severe asthma as well as nocturnal asthma phenotypes are frequently associated with concurrent sleep breathing disorders and therefore present intermittent hypoxic events (Ross et al., 2012; Broytman et al., 2015).

### Carbon Dioxide

CO_2_ is the companion gas of O_2_ in the respiration process, and as such CO_2_ is similarly affected by pathologic situations in the case of respiratory diseases (reduction in alveolar ventilation or in lung gas diffusion) or in cancer (abnormal blood circulation in tumors), promoting both hypoxia and hypercapnia/acidosis at both tissue and cellular levels. However, although it is known that the CO_2_ partial pressure in the cell microenvironment specifically depends on tissues and pathophysiological conditions, almost all research on cell cultures to date has been conducted at a concentration of 5% CO_2_, using buffers to restrict the pH to a 7.4 standard value. Furthermore, reports studying hypercapnia have been conducted at higher CO_2_ partial pressures, which do not necessarily correspond to those experienced *in vivo* by the studied cells. Although modifying the cell culture pH is readily implemented (Apkon et al., 1997; Müthing et al., 2003; Park et al., 2012; Kim et al., 2016; Boussadia et al., 2018), care should be taken to avoid potential confounding effects of pH on the activity of drugs employed in the medium (Jha et al., 2014; Swiech et al., 2016; Fontaine et al., 2017; Wendelboe et al., 2017).

In the case of research on malignant tumor cell cultures, realistically controlling extracellular CO_2_ partial pressure is of interest, given the important role that hypercapnia/acidosis play in the pathophysiology of tumor proliferation, invasiveness, and metastasis (Obata et al., 2013; Hulikova et al., 2014; Swietach et al., 2014; Corbet and Feron, 2017). Sleep apnea is a disease in which cells *in vivo* are subjected not only to intermittent hypoxia, but also to intermittent hypercapnia. However, most cell and animal models that studied the mechanisms involved in OSA exclusively focused on the hypoxic challenges and ignored the CO_2_ perturbations. Nevertheless, recent sleep apnea studies showing that hypercapnia accelerates adipogenesis (Kikuchi et al., 2017) or is more important than hypoxia in neural outcomes (Wang D. et al., 2016) or can critically modulate ventilatory long-term facilitation (Deacon et al., 2017) have strengthened the interest of including both hypoxic and hypercapnic challenges in sleep apnea research. Such combined challenges have only scarcely been applied in animal models (Xue et al., 2017; Tripathi et al., 2018), but has yet to be explored in realistic cell culture models. Mechanical ventilation is a frequently used treatment modality in which CO_2_ plays a significant role. Since the initial proposal of the concept of protective hypercapnia to reduce ventilator-induced lung injury, decades ago (Hickling et al., 1990), considerable clinical research has been carried out to analyze the potential benefits and drawbacks of allowing increased levels of CO_2_ partial pressure in patients with acute respiratory failure, as recently reviewed for both adult and pediatric patients (Barnes et al., 2017; Reiterer et al., 2017). However, information on how well-controlled different levels of CO_2_ partial pressure and acidosis at cell level modify the pathophysiological mechanisms as well as information on the outcome of such approaches is currently dismally scarce.

### Other Gases

Notwithstanding O_2_ and CO_2_, other gases also play important roles in modulating cell behavior and as such have been proposed as potential therapeutic agents, in particular the gasotransmitters CO, NO, and H_2_S (Wang, 2014). At sufficiently low doses, the potentially dangerous carbon monoxide is cyto- and tissue-protective in models of sepsis and acute lung injury, currently being tested as a therapeutic agent for inflammatory and proliferative diseases (Ryter et al., 2018). Nitric oxide is a potent endogenous vasodilator that can be exogenously administered via inhalation, to provide selective pulmonary vasodilatation in well-ventilated lung units, to improve ventilation-perfusion mismatch, and to reduce pulmonary vascular resistance and pulmonary hypertension in acute lung injury or other conditions associated with pulmonary vasoconstriction (Wu et al., 2014; Asadi et al., 2015; Hajian et al., 2016; Helms et al., 2018). The mechanisms involving NO biological activity are currently quite well understood and this gas has already been tested in adult and pediatric clinical trials (Gebistorf et al., 2016). Hydrogen sulfide is an endogenously produced gas and participates in the physiological regulation of several tissues and organs. Research in different animal and cell models using H_2_S donors has shown therapeutic potential for a variety of diseases, such as arterial and pulmonary hypertension, atherosclerosis, ischemia–reperfusion injury, or heart failure (Bełtowski, 2015; Perry et al., 2018). Another simple gas that, in addition to gasotransmitters, has been proposed as a therapeutic agent is molecular hydrogen. H_2_ should operate as an antioxidant by reacting with highly reactive oxidants, such as hydroxyl radical and peroxynitrite inside cells. Different tests, such as inhaling H_2_ gas, drinking H_2_-dissolved water, or injecting H_2_-dissolved saline, have been tested in both animal and patient models and positive results have preliminarily been reported (Ohta, 2015).

So far, most research on gases other than O_2_ and CO_2_ has been conducted, in both animal models and in patients, by administering the gas through inhalation (CO, NO, H_2_), water solution (H_2_), or by administering donor molecules (H_2_S) and in some cases by providing a combination of two of these gases (Liu et al., 2015; Nishida et al., 2018). Although it is possible to measure the gas concentration in the blood, the actual concentration value at the cell level in different tissues and organs is unknown. Therefore, to further our understanding on the pathologic mechanisms involved and on the therapeutic levels required, *in vivo* research should be carried out, under well-controlled gas concentrations aimed at the cell microenvironment.

## Limitations of the Conventional Settings to Modify Gas Partial Pressure in Cell Cultures

The most simple and straightforward method to subject cultured cells to a given gas concentration consists of setting the partial pressure of the gas on top of the culture media to the desired value. Theoretically, after reaching the equilibrium between the gas and liquid phases, the concentration in the cell medium equals that of the gas phase. However, this commonly used procedure does not ensure that the cell microenvironment at the bottom of the liquid phase achieves the desired gas partial pressure, since accomplishment of the implicit hypotheses of homogeneity and stationarity is not warranted and is in fact highly unlikely.

Some of the limitations of the conventional setting for subjecting cultured cells to specific gas concentration have been clearly enunciated and reported (Allen et al., 2001). Allen et al. (2001) used a Clark oxygen electrode to measure the actual partial pressure of O_2_ within the culture medium when modifying the concentration of the gas in the air phase. A relevant result was that the time required to fully equilibrate the medium with the external oxygen concentration was >3 h in most experiments (Allen et al., 2001). Such long equilibration times are not unexpected from the Fick’s law when applied to oxygen diffusion from the gas–liquid interface to the bottom of the culture plate. Indeed, taking into account that the coefficient of diffusion (*D*) of O_2_ in water at 37°C is 2.6 × 10^-5^ cm^2^/s (Han and Bartels, 1996) and that, according to Einstein’s equation, the expected time (*t*) to reach a 3D diffusion distance (*d*) is *t* = *d*^2^/(6*D*), a time of ∼1.8 h would be expected for a diffusion travel distance of 1 cm. Similar results would be obtained for CO_2_ since this gas has a coefficient of diffusion in water remarkably close to that of O_2_ (Vaupel, 1976).

**FIGURE 2:**
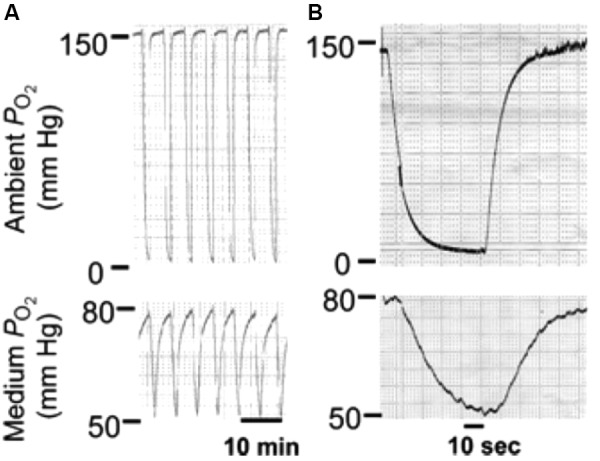
Conventional setting. Changes in oxygen partial pressure (PO_2_) in the tissue culture chamber and the medium during application of intermittent hypoxia (IH). **(A)** Representative tracings of PO_2_ during IH in the tissue culture chamber (top panel) and the medium near the cells (bottom panel). **(B)** Expanded tracing of the PO_2_ changes during a single episode of IH. Reproduced from Yuan et al. (2004) with permission of the copyright owner.

Long diffusion times required for equilibrating gas concentrations across the whole culture medium should not be a great problem in case the aim is to keep cultured cells at a given gas partial pressure for several hours or days. Nevertheless, slow equilibration times pose a difficulty when cells must be subjected to intermittent hypoxia at the fast rate of changes typical of sleep apnea or mechanical ventilation (Figure 1). The conventional setting is therefore unable to provide intermittent hypoxia to cultured cells at the pathophysiological frequency of interest in sleep apnea (up to 60 cycle/hour), as it is only possible to achieve much slower intermittent hypoxic rates, which are not satisfactory for mimicking patients with sleep apnea. For instance, it was reported that applying cycles consisting of 15 s at 1.5% O_2_ followed by 4 min at 21% O_2_ in the gas phase resulted in culture intermittent hypoxia ranging from 11% to 7% (equivalent to 15 events/h) (Yuan et al., 2004; Figure 2). Other studies using the conventional setting allowed intermittent hypoxia cycling rates below severe sleep apnea (e.g., 12 event/h) (Polotsky et al., 2010). However, there are no data showing that this experimental approach can actually apply the ∼60 cycles/h of intermittent hypoxia to the cell microenvironment. It seems therefore impossible that the conventional setting could subject cells to the much faster rates of intermittent hyperoxia (∼300 cycles/h) induced by mechanical ventilation in some cases (Figure 1).

In addition to the considerable problem of the response times required to achieve the desired oscillation in cellular gas concentrations, there is also another issue concerning culture medium homogeneity that should be considered when evaluating the conventional setting for subjecting cultured cells to well-controlled gas partial pressures. On the one hand, the partial pressure achieved at the bottom of the culture chamber, i.e., cell microenvironment, under stationary conditions does not necessarily equal the one in the air phase. Indeed, one should take into account that cells are actively modifying gas concentrations, particularly in the case of respiratory gases, since they take O_2_ from the medium and introduce CO_2_ back to the medium. Oxygen consumption and CO_2_ production modify the equilibrium conditions by creating a vertical gradient of gas concentrations from the cell layer to the gas–liquid interface. In other words, if the steady state has been achieved, the partial pressure of each of the gases at the bottom of the cell culture chamber is markedly different when there are cells in the culture dish as opposed to a medium only (Allen et al., 2001). The magnitude of the spectrum range differences would depend on the number and type of cultured cells and on their respiratory activity, which is difficult to predict in most specific experimental conditions. On the other hand, it should be mentioned that the liquid in a culture dish is not static either, but spontaneously circulates in a radial pattern, even when the dish is at rest, thereby establishing a *de facto* convection flow within the medium (Lindsay and Yin, 2016), consequently making it possible to induce radial (horizontal) non-homogeneity in gas partial pressures. Whereas such liquid convection may contribute to reduce vertical gas concentration gradients within the culture medium, their specific effects in modifying the actual gas pressure at the cell microenvironment are *a priori* unknown.

The fact that the gas partial pressure actually experienced in the culture cell microenvironment is affected by the previously mentioned non-stationarity and non-homogeneity issues makes it unpredictable to exactly what level of gas concentration cells will be exposed to, in any given experimental setting. The possibility of measuring the actual levels of gas partial pressure at the bottom of the culture plate by means of small size sensors (Yuan et al., 2004; Polotsky et al., 2010; Tsapikouni et al., 2012) seems relatively unfeasible for routine experiments. Developing and using methodologies to accurately control gas concentrations to which the cells are subjected, both under static and dynamic conditions, are therefore required.

## Optimized Control of Gas Partial Pressure in Cultured Cells

### Circulation of Gas-Preconditioned Culture Medium

**FIGURE 3:**
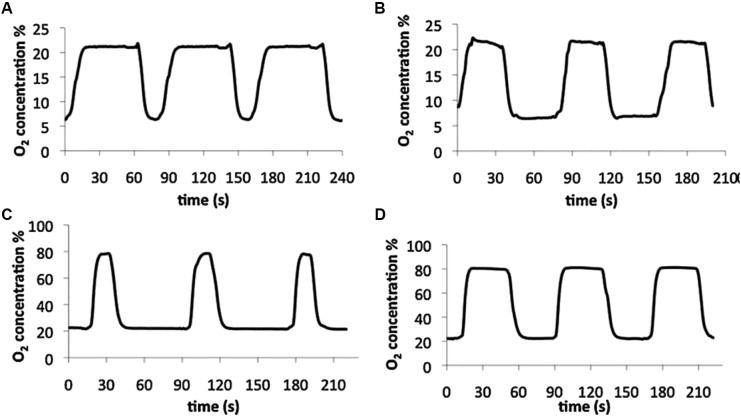
Experimental setting based on circulating gas-preconditioned culture medium. Examples of measured oxygen concentration patterns in cell levels obtained with four settings of the designed bioreactor: intermittent hypoxia (**A** and **B**) and intermittent hyperoxia (**C** and **D**). Reproduced from Tsapikouni et al. (2012) with permission of the copyright owner.

Circulating a gas-preconditioned culture medium to ensure that cells are always in contact with a liquid with the target gas partial pressure is an approach that should precisely control gas partial pressure within the cultured cell microenvironment. The initial setting was designed for applying intermittent hypoxia mimicking sleep apnea (Baumgardner and Otto, 2003) and was based on using forced medium convection to induce reproducible mixing of the medium, while cycling media containing different O_2_ concentrations in cycles lasting several seconds. This setting was able to realistically apply intermittent hypoxia ranging ∼0–80 mmHg of O_2_ partial pressure with a change time constant of less than 2 s. However, cells were cultured inside capillary tubes which made it difficult to carry out some of the most conventional cell biology techniques, such as optical and fluorescent microscopy. A further development of the concept of circulating gas-preconditioned media on the cells consisted of a setting based on exposing cells to a medium that is bubbled with the appropriate mixture of gases into two separate containers and directed to the cells being cultured in standard glass coverslips with the aid of bidirectional peristaltic pumps (Tsapikouni et al., 2012). This experimental setting allowed actual application of intermittent hypoxia/hyperoxia–normoxia at rates corresponding to severe sleep apnea (60 cycles/h) (Figure 3) with the advantage that the cell culture is readily available for inspection with any microscopy technique or bio-analytical assay.

However, settings based on circulating the cell medium have a potential drawback, since the medium flowing on the cell layer induces shear stress, which is a well-known physical challenge inducing marked cell responses, particularly in blood vessel-derived cells and tumor cells (Li et al., 2005; Huang et al., 2018). The magnitude of the shear stress would depend on the velocity of the medium and on the total time of the circulatory cycle. Whereas shear stress would be negligible for quasi-static and low-frequency intermittent gas concentrations, it may add a major confounding factor in case of experimental settings mimicking conditions requiring relatively high-amplitude high-frequency medium flow such as intermittent hypoxia or mechanical ventilation (Figure 1). In these cases, control experiments under normoxia/normocapnia could be carried out to evaluate the sole effects of shear stress, but care should be taken in the interpretation, since the possible effects of a double-hit (cyclic hypoxia/hypercapnia plus simultaneous shear stress) challenge effect should not be discarded. In fact, it has recently been found that low shear stress induces endothelial reactive oxygen species (Chao et al., 2018), thereby potentially contributing to the oxidative stress response induced by intermittent hypoxia/hyperoxia.

### Gas Diffusion Through a Thin Membrane

**FIGURE 4:**
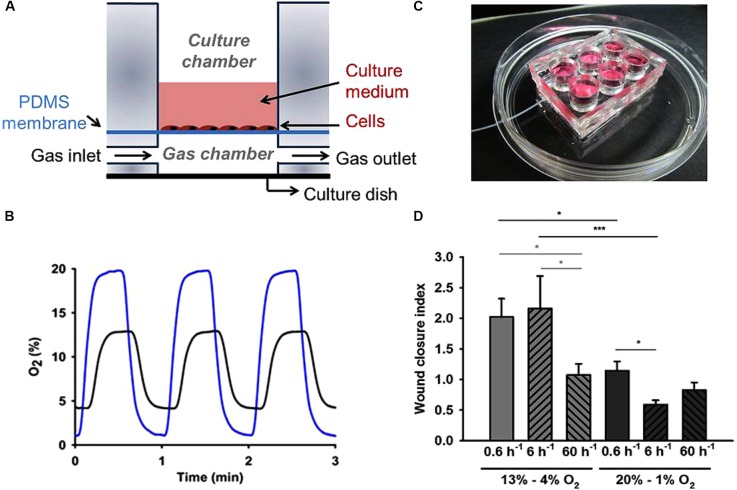
Experimental setting based on gas diffusion through a thin gas permeable membrane. **(A)** Schematic view of a cell culture well based on a gas permeable membrane. Gas partial pressure applied to the cells cultured on one side of the membrane is virtually the same as in the gas circulating through the other side of the membrane. **(B)** Example of intermittent hypoxia (IH) patterns actually measured on top of the membrane (cell culture level) when applying IH with different magnitudes [20–1% O_2_ (blue line) and 13–4% O_2_ (black line) at a frequency of 60 cycles/h]. **(C)** Real image of a setting containing six wells. **(D)** Human aortic endothelial cells wound closure index under IH. Both frequency and amplitude range of IH induced significant changes in wound healing. Low-frequency IH (0.6 cycles/h) accelerated endothelial wound healing in the 4–13% magnitude range, while high-frequency IH patterns simulating severe OSA (60 cycles/h) did not significantly modify wound closure rates regardless of oxygen levels. Data were normalized to the condition of continuous 13% O_2_. *n* = 6 each group. ^∗^*P* < 0.05; ^∗∗∗^*P* < 0.001. **(A)** and **(C)** reproduced from Campillo et al. (2017a) (http://creativecommons.org/licenses/by/4.0/). **(B)** and **(D)** reproduced from Campillo et al. (2017b) with permission from the copyright owner.

A more straightforward approach to control the gas partial pressures *in vitro* is based on culturing cells on one side of a thin membrane permeable to the gas of interest and circulating the air with the desired gas partial pressure on the other side of the membrane. In this way, the fast gas diffusion time through the thin membrane allows subjecting the cell layer to the targeted gas concentrations, as shown by the diagram in Figure 4A. Polydimethylsiloxane (PDMS) is a very convenient material for such membranes because it is biocompatible, has good gas permeability, and is optically transparent (Ni et al., 2009). PDMS membranes of different widths are commercially available or can be readily fabricated in the laboratory (Campillo et al., 2016) and then coated with extracellular matrix proteins such as collagen or fibronectin for cell culture. Given that PDMS has a coefficient of oxygen diffusion *D* = ∼3.5 × 10^-5^ cm^2^/s (Charati and Stern, 1998; Merkel et al., 2000) which is slightly greater than water (2.6 × 10^-5^ cm^2^/s), using a sufficiently thin membrane ensures that cells on one side of the membrane are indeed subjected to a well-controlled gas partial pressure (virtually the same as in the gas on the other side of the membrane). For instance, a 100 μm width membrane (*D*) presents a diffusion time (*t* = *d*^2^)/(6*D*) of ∼0.5 s, which ensures almost instantaneous transmission of gas partial pressure from the gas into the cell microenvironment, as reflected by the intermittent hypoxia pattern measured at the cell level, as shown in Figure 4B. Interestingly, PDMS has very similar permeability for different gases of biological interest (Charati and Stern, 1998; Merkel et al., 2000).

Thin PDMS membranes were used in different settings to apply controlled oxygen partial pressures to cultured cells (Oppegard et al., 2009; Brennan et al., 2015; Campillo et al., 2017a; Minoves et al., 2017) and to study the cross-talk between cells simultaneously exposed to different oxygenation profiles mimicking the hypoxic gradients within tumors (Almendros et al., 2017). Although the PDMS membrane is very thin for such applications, its surface can correspond to conventional culture wells (Figure 4C). Moreover, given that liquid PDMS can be easily molded and cross-linked in the laboratory, settings consisting on different gas-permeable wells can readily be obtained for simultaneous experiments (Campillo et al., 2017a) as shown in Figure 4C. Interestingly, having such wells a conventional culture area allows cells under controlled gas conditions to be subjected to all biological and molecular common tests, including those involving real-time microscopic cell inspection. As an example, Figure 4D shows the results obtained in wound healing assays when subjecting human aortic endothelial cells to patterns of intermittent hypoxia with different amplitudes and frequencies (Campillo et al., 2017b). This example clearly illustrates the advantages and scientific relevance of subjecting cells to realistic values of oxygenation. Indeed, when cells were subjected to 13–4% O_2_ swings, which are realistic in aortic endothelium in sleep apnea, wound healing at very low frequency (0.6 cycles/h) was twofold versus at normal oxygenation levels (13% O_2_). When cycling frequency was similar to those in patients with severe sleep apnea (60 cycles/h), repair was similar to normoxia, suggesting that fast hypoxia cycling does not necessarily imply a higher cell response. Interestingly, wound healing decreased for non-physiologic hypoxic 20–1% O_2_ swings (Figure 4D).

### Microfluidic Devices to Control Gas Partial Pressure in Cultured Cells

The concepts of a circulating medium and using thin gas-permeable membranes can be implemented in macroscopic settings with cell culture surfaces similar to the ones in a conventional culture, as shown above. Moreover, they can also be implemented in microscopic settings using microfluidic techniques to obtain chips for specific control of gas partial pressures in cultured cells (Brennan et al., 2014). This alternative technique has its advantages but also has some drawbacks (Halldorsson et al., 2015). One advantage is that using sophisticated fabrication techniques, chips can be designed to mimic cell microenvironments to optimally fit different applications. For instance, microfluidic devices were developed to study cancer metastasis under chronic and intermittent hypoxia (Acosta et al., 2014), to mimic radial biological gradients (Li et al., 2015) or to investigate cell migration under perpendicular chemical and oxygen gradients (Chiang et al., 2017). Microfluidic-based chip fabrication also allows for superimposing intermittent hypoxia to modulated glucose flow in a setting mimicking pancreatic islets (Lo et al., 2012). Another obvious advantage of microfluidic settings is that, given their size, they are of potential interest for high-throughput applications in genomics or proteomics studies and in drug testing. However, microfluidic systems have two major drawbacks in their use in conventional labs. First, given their complexity, they can only be fabricated in specialized research facilities or industries. Second, common laboratory techniques, e.g., supernatant and scrapped cells analysis and direct microscopic cell observation, can be hindered by the reduced size and geometry available for cultured cells.

**FIGURE 5:**
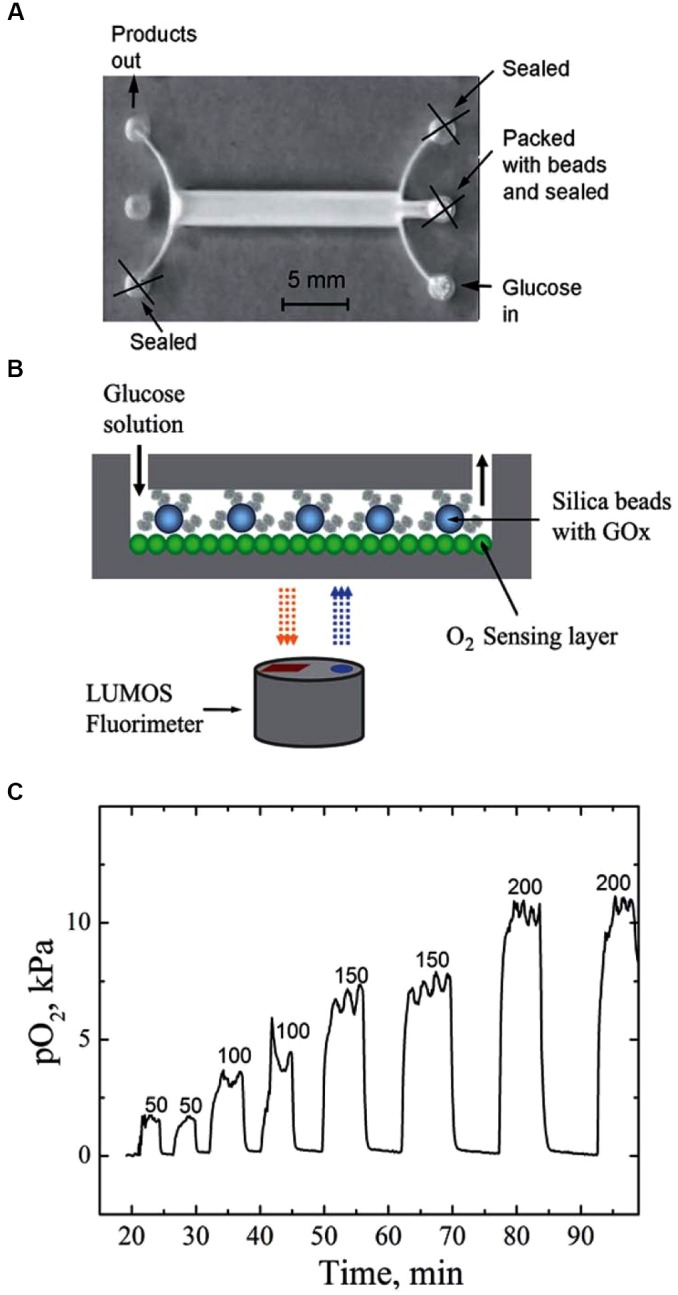
Microfluidic system integrating a fluorimetric oxygen sensor based on O_2_-sensitive nanoparticles. **(A)** Photographic image of the reactor; **(B)** schematic representation of the experimental setup; **(C)** real-time monitoring of oxygen concentration (PO_2_) inside the reactor at different flow rates (ml min^-1^) when monitoring of cell enzymatic activity. Reproduced from Lasave et al. (2015) with permission of the copyright owner.

It is interesting to note that in addition to controlling the gas partial pressures applied to cells, microfluidic devices may incorporate microsensors that are able to measure different physiological variables, in particular gas concentration and pH at the cell site, in real time, without cell perturbation (Modena et al., 2018). For instance, a microfluidic device coupled with a micro-fabricated Clark-type oxygen electrode was used to measure changes in the respiratory activity of *Escherichia coli* bacteria co-cultured with IFN-g-activated or non-activated neutrophil-like cells. Using that system, the authors were able to demonstrate that the rate of elimination of *E. coli* increased as the activity of the neutrophil-like cells increased (Yamagishi et al., 2017). Oxygen sensors based in an optical technology can also be incorporated into cultured cells. Indeed, the cell culture surface can be coated with conjugated polymeric nanoparticles containing a covalently grafted oxygen indicator. The signal of the integrated sensor—with a response time of 0.2 s—can be read-out using unsophisticated, compact, and low-cost RGB/NIR cameras (Lasave et al., 2015). Figure 5 illustrates the capability of such a setting to continuously monitor an enzymatic conversion in silica beads that contain immobilized glucose oxidase, which showed that the system was very sensitive to changes in the flow rate of the glucose solution. The observed plateaus resulted from the balance between O_2_ injected with the air-saturated and the consumption O_2_ by the enzymatic reaction (Lasave et al., 2015).

Advanced microfabricated cell culture settings incorporating sensors for oxygen and other relevant physiological variables are currently under development, and are progressing at a fast pace (Kieninger et al., 2018b; Kilic et al., 2018; Zirath et al., 2018). It is therefore expected that in addition to those devices already commercially available (Kieninger et al., 2018a), a large variety of commercial cell culture systems will soon become readily available which will hopefully drive their cost down.

## Conclusion

Cell cultures should ideally be carried out in conditions reproducing those within the native extracellular microenvironment. Given that most tissues are 3D and have soft surfaces rather than hard ones, along with O_2_ concentrations typically below 19%, it seems that the most common *in vitro* setting consisting of seeding cells on 2D rigid plastic/glass wells, within a physiologically hyperoxic culture chamber, is a far cry from mimicking the natural cell niche environment. Specifically focusing on O_2_, CO_2_, and gasotransmitters that modulate important pathophysiological mechanisms, cultured cells should be subjected to gas concentrations with magnitudes and fluctuation rates corresponding to the disease model under study. To this end, optimized experimental settings which overcome the limitations of the conventional cell culture procedure are required. Excellent performance in statically and dynamically controlling gas partial pressure at the cultured cell microenvironment can now be achieved by using thin gas-permeable PDMS membranes. This procedure can be incorporated into sophisticated and high-performance microfluidic systems. Interestingly from a practical perspective and for allowing widespread application, PDMS membrane-based settings for optimally controlling gas concentrations can also be easily assembled in any conventional cell biology laboratory.

## Ethics Statement

No experiments in cells, animals, or humans have been performed to write this review. Accordingly, no Ethics Board permission was required.

## Author Contributions

RF conceived and drafted this review. IA, JM, DG, and DN actively participated by providing their methodological, physiological, and clinical perspectives on the topic, and significantly contributed to the final version.

## Conflict of Interest Statement

The authors declare that the research was conducted in the absence of any commercial or financial relationships that could be construed as a potential conflict of interest.
